# D-glyceric aciduria due to GLYCTK mutation: Disease or non-disease?

**DOI:** 10.1016/j.ymgmr.2024.101159

**Published:** 2024-11-12

**Authors:** Sandra D.K. Kingma, Laura K.M. Steinbusch, Sietse M. Aukema, Margje Sinnema, Bianca Panis, Joost Nicolai, Estela Rubio-Gozalbo

**Affiliations:** a Mosakids Children's Hospital, Maastricht University Medical Centre+, P. Debyelaan 25, P.O. Box 5800, 6202 AZ Maastricht, The Netherlands; b Centre for Metabolic Diseases, University Hospital Antwerp, University of Antwerp, Drie Eikenstraat 655, 2650 Edegem, Antwerp, Belgium; c Department of Clinical Genetics, Maastricht University Medical Centre+, P. Debyelaan 25, P.O. Box 5800, 6202 AZ Maastricht, The Netherlands; d Department of Neurology, Maastricht University Medical Centre+, P. Debyelaan 25, P.O. Box 5800, 6202 AZ Maastricht, The Netherlands

**Keywords:** D-glyceric aciduria, GLYCTK, D-glycerate-2-kinase deficiency

## Abstract

D-glyceric aciduria (DGA) is caused by D-glycerate-2-kinase deficiency due to biallelic pathogenic variants in *GLYCTK.* It is associated with variable neurological symptoms. DGA is extremely rare, and genetic variants are only known in 7 previously described patients. We report a new patient with DGA and a novel homozygous *GLYCTK* variant.

## Highlights

• D-glyceric aciduria is a rare genetic disorder that may be severely underdiagnosed.
• The clinical significance of D-glyceric aciduria is yet uncertain due to the broad clinical spectrum.
• We describe the 8th genetically confirmed case of D-glyceric aciduria in which no other cause for disease manifestations could be identified.

## 1 Introduction

D-glyceric aciduria (DGA; OMIM #220120) was firstly described in 1974 by Brandt et al. [1]. Since then, only 16 new patients were described. In 7, the genetic variants are known [2,3]. DGA is caused by biallelic variants in *GLYCTK* ([Genbank NG_023246]). Patient characteristics vary from normal development to epileptic encephalopathy, speech delay, severe hypotonia and autism [2]. We describe a rare new case of DGA, caused by a novel homozygous *GLYCTK* variant.

## 2 Patient description

A 7-year-old boy presented with intellectual disability and incontinence for stools. He had delayed speech, short attention span, repetitive movements, clumsiness and abnormally brisk reflexes. At 8 years, he had developed sensorineural hearing loss. He was the fourth child of Moroccan parents. Consanguinity could not be excluded according to the parents: they were from the same small village in Marocco. Mother and a brother suffered from sensorineural hearing loss. Another brother had autistic like behavior, speech delay and a clumsy rigid movement pattern. He refused testing. The sister had delayed speech.

Magnetic Resonance Imaging (MRI) and Electro-encephalogram both showed no abnormalities.

Targeted Urine Metabolomics (TUM) [4], which was used as the initial screening approach, showed increased D/L-glyceric acid excretion. Glyceric acid excretion is increased in DGA and in hyperoxaluria II (L-glyceric aciduria; OMIM #260000) [2]. Since oxalic acid excretion was normal, DGA was suspected. Subsequently, D-glyceric acid was quantified in both available urines using HPLC-QTOFMS (6343 and 8812 μmol/mmol creatinine, normal range: 0.4–12 μmol/mmol creatinine). The remainder of the extensive metabolic screening in plasma and urine was normal (transferrin isoelectric focusing, neuraminic acid, acylcarnitines, amino-acids, very-long-chain-fatty-acids, homocysteine, methylmalonic acid, and urinary guanidinoacetate, glycosaminoglycans, oligosaccharides, neuraminic acid). An exome based metabolic gene panel (732 genes) identified a homozygous variant (c.853 A > T; p.(Lys285*)) in *GLYCTK,* predicted to result in a truncated protein, removing approximately 45 % of the protein. As this variant was localized in the last exon, it likely escaped nonsense-mediated decay. Both parents were carrier. Applying (modified) ACMG-criteria, the variant was classified as likely pathogenic [5]. Because of the heterogeneous phenotype of DGA, whole exome sequencing trio-analysis was extended with an intellectual disability panel (1612 genes) including an exome wide copy number variant (CNV) analysis, in addition to a single nucleotide polymorphism (SNP) array and *FMR1*-analysis, but were normal. The hearing impairment seemed to be an individual clinical entity, because it is not associated with DGA, and there were several family members suffering from hearing impairment that did not share the other clinical characteristics of our patient. An exome based hearing impairment gene panel (256 genes) identified a homozygous pathogenic variant (c.4837G > T; p.Glu1613*) in the *STRC* gene that explained the hearing impairment.

## 3 Discussion

**Fig. 1:**
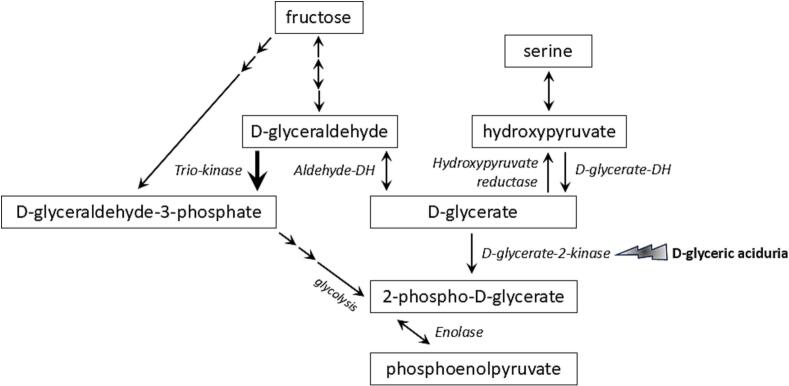
Schematic illustration of the relation between fructose, serine and D-glycerate metabolism. Lightning represents the enzyme deficiency in D-glyceric aciduria. Multiple arrows represent multiple steps. Double-arrowed lines represent bidirectional steps. Bold arrow represents dominant step. DH; dehydrogenase. Image is based on reference [8, 9, 23].

D-glyceric acid (Fig. 1) is an intermediate of serine and fructose metabolism [6,7]. It is converted to 2-phosphoglycerate by the enzyme D-glycerate-2-kinase (EC 2.7.1.165) encoded by *GLYCTK* [[8], [9], [10]]. Confusingly, other literature [11] and databases such as Uniprot report the conversion of D-glyceric acid to 3-phosphoglycerate by the enzyme glycerate-3-kinase (EC:2.7.1.31) also encoded by *GLYCTK*. All these databases and literature agree that a variant in *GLYCTK* can result in D-glyceric aciduria. Zehavi et al. [2] reviewed 16 published cases of DGA in 2019, and afterwards only one additional patient has been reported [3]. DGA might be severely underdiagnosed because in the past, the measurement of urinary organic acids had a low sensitivity for glyceric acid [12,13]. Nowadays, biochemical analysis of D/L-glyceric acid is integrated in external quality assurance schemes (for instance ERNDIM quality control) and the performance for D/L-glyceric acid is good. The enzyme D-glycerate-2-kinase has been reported as unstable [12,14] and can only be measured in liver tissue or cell models [12,15]. The potential pathophysiology of the DGA is unknown. However, several metabolic processes can be impacted by fructose or glycerate: 1) glycolysis and the citric acid cycle (Krebs cycle), since the downstream metabolite of D-glyceric acid, 2-phospho-D-glycerate, is a glycolysis intermediate. 2) In the central nervous system, elevated fructose and glycerate levels could disrupt the synthesis of neurotransmitters, particularly those involved in excitatory and inhibitory signaling (e.g., glutamate and GABA). Fructose has been associated with neuro-inflammation and oxidative stress with a negative impact on neuroplasticity [16]. 3) High glycerate from intestinal fructose metabolism induced islet cell damage and glucose intolerance in a murine model that received a high-fat-diet [17]. The importance of this pathway in humans can be debated since no DGA patients with glucose intolerance have been described.

Some therapeutic diets have been tried. In several patients, D-glyceric acid excretion was shown to be highly dependent on dietary fructose. Oral loading tests with fructose and serine resulted in increased D-glyceric acid excretion [13,14,18,19]. Some patients on a fructose-restricted diet showed normalization of DGA and neurological improvement [19,20]. One patient showed no improvement of DGA and neurological manifestations after a fructose- and protein-restricted diet, but was only treated during several days [15]. Another patient had metabolic acidosis and was chronically treated with bicarbonate [13]. Some patients, as our patient, never received diet or medication [3]. Evidence of effectiveness of the interventions is very limited while it poses an extra burden for the patient.

DGA has been associated with a wide phenotypic spectrum. Some patients show moderate intellectual disability, speech delay and autistic behavior, such as our patient. Some suffer from progressive epileptic encephalopathy and spastic quadriparesis [2].

Some authors hypothesized that DGA is a non-disease. Largillière et al. [21,22] described siblings with encephalopathy, failure to thrive and spastic tetraplegia. However, only one sibling had DGA. Another congenital disease was suspected but not identified [21,22]. Some DGA patients with neurological deficits had siblings with DGA that were non-symptomatic [18,23]. Other authors hypothesized that this may be caused by yet unidentified modifying genetic factors that influence the phenotype [2]. Finally, some patients showed comorbidities that may have caused or contributed to the clinical phenotype. One patient had both genetically confirmed DGA and nonketotic hyperglycinemia [1,24,25]. Another DGA patient showed pituitary deficiency [3,12]. A third patient exhibited decreased enzyme activity of respiratory-chain-complexes I/IV, which may have also been partly due to valproic acid use [2].

Consanguinity in many of the families makes it difficult to assess if DGA is the cause of the symptoms due to the possibility of other recessive diseases. *GLYCTK* is, however, a highly conserved gene, which suggests an important role [3]. In most patients, no other cause could be identified. Most patients have been described years to decades ago, but in more recently described patients, extensive metabolic and/or genetic screening was performed. Because its pathophysiology is unknown, and asymptomatic patients have been described, other possible (genetic) causes or contributors should be investigated in DGA patients.

## 4 Conclusions

DGA is an extremely rare metabolic disorder. We described the 8th patient in which a genetic cause has been identified. Despite extensive metabolic and genetic testing, no other explanation for the phenotype could be identified. Therefore, we tentatively hypothesize that DGA might indeed be a metabolic disorder with variable expression that, in combination with other not yet identified factors, may lead to a potentially devastating neurological phenotype. It is important that the natural history and genetic variants of these patients are reported. Also, more research on the pathophysiological mechanisms, potential modifiers and treatment options is needed. Counseling of these families remains challenging.

## Author statement

Ethics approval was not necessary because it is a short report of a case.

We did not receive funding for this project.

## CRediT authorship contribution statement

**Sandra D.K. Kingma:** Writing – review & editing, Writing – original draft, Visualization, Investigation, Conceptualization. **Laura K.M. Steinbusch:** Writing – review & editing, Supervision, Investigation, Formal analysis. **Sietse M. Aukema:** Writing – review & editing, Investigation, Formal analysis. **Margje Sinnema:** Writing – review & editing, Investigation. **Bianca Panis:** Writing – review & editing, Supervision, Investigation. **Joost Nicolai:** Writing – review & editing, Investigation. **Estela Rubio-Gozalbo:** Writing – review & editing, Visualization, Supervision, Investigation, Conceptualization.

## Declaration of competing interest

Sandra Kingma has no competing interests.

Bianca Panis has no competing interests.

Estela Rubio-Gozalbo has no competing interests.

Joost Nicolai has no competing interests.

Sietse Aukema has no competing interests.

Margje Sinnema has no competing interests.

Laura Steinbusch has no competing interests.

Trial registration: not applicable.

The parents of the patient gave informed consent for this study and publication.

## Data Availability

No data was used for the research described in the article.
